# Expert Opinions on Improving Femicide Data Collection across Europe: A Concept Mapping Study

**DOI:** 10.1371/journal.pone.0148364

**Published:** 2016-02-09

**Authors:** Carmen Vives-Cases, Isabel Goicolea, Alison Hernández, Belen Sanz-Barbero, Aisha K. Gill, Anna Costanza Baldry, Monika Schröttle, Heidi Stoeckl

**Affiliations:** 1 Department of Community Nursing, Public Health and Preventive Medicine and History of Science, Alicante University, Alicante, Spain; 2 CIBER de Epidemiología y Salud Pública (CIBERESP), Barcelona, Spain; 3 Public Health Research Group, Alicante University, Alicante, Spain; 4 Epidemiology and Global Health Unit, Department of Public Health and Clinical Medicine, Umeå University, Umeå, Sweden; 5 Escuela Nacional de Sanidad, Instituto de Salud Carlos III, Madrid, Spain; 6 Department of Social Sciences, University of Roehampton, London, United Kingdom; 7 Department of Psychology, Second University of Naples, Naples, Italy; 8 Department of Rehabilitation Science, Technical University of Dortmund, Dormund, Germany; 9 Department of Global Health and Development, London School of Hygiene and Tropical Medicine, London, United Kingdom; St Francis Hospital, UNITED STATES

## Abstract

Femicide, defined as the killings of females by males because they are females, is becoming recognized worldwide as an important ongoing manifestation of gender inequality. Despite its high prevalence or widespread prevalence, only a few countries have specific registries about this issue. This study aims to assemble expert opinion regarding the strategies which might feasibly be employed to promote, develop and implement an integrated and differentiated femicide data collection system in Europe at both the national and international levels. Concept mapping methodology was followed, involving 28 experts from 16 countries in generating strategies, sorting and rating them with respect to relevance and feasibility. The experts involved were all members of the EU-Cost-Action on femicide, which is a scientific network of experts on femicide and violence against women across Europe. As a result, a conceptual map emerged, consisting of 69 strategies organized in 10 clusters, which fit into two domains: “Political action” and “Technical steps”. There was consensus among participants regarding the high relevance of strategies to institutionalize national databases and raise public awareness through different stakeholders, while strategies to promote media involvement were identified as the most feasible. Differences in perceived priorities according to the level of human development index of the experts’ countries were also observed.

## Introduction

“Femicide” is becoming recognized worldwide as the ultimate manifestation of violence against women and girls [1]. Diana Russell proposed the term for the first time at the International Tribunal on Crimes Against Women in 1976 in order to name the intentional “killings of females by males because they are females”[2], and it is now broadly used also at the UN level [3]. In relation with this definition of Femicide, different forms of women’ killing are recognized such as intimate partner-related killings [4], so-called “Honour”[5] and Dowry-related murders, forced suicide [6], female infanticide [7] and gender-based sex-selective foeticide [8], genital mutilation-related death cases [9], targeted killing of women at war [10] and in the context of organized crime, among others [11]. A broader definition of this term also refers to the responsibility of States in women’s death-cases perpetuated by misogynous attitudes and/or social discriminatory practices against women. In this broader definition are included, for example, deaths associated to lack of accessibility to healthcare for women and girls, gender-based selective malnutrition and trafficking of women for prostitution or drugs [12]. As it is difficult to decide in all cases of killings of women and girls if they had been killed because of their gender, researchers often include all killings of women in the first step and then differentiate between forms that are more or less relevant with regard to gendered backgrounds and motives. This is also a reason why consensual research on prevalence and nature and characteristics of victims and offenders and their relationship is needed.

Approximately 66,000 women every year from 2004 to 2009 were victims of killings, representing almost one-fifth of total homicide victims (396,000 deaths) in an average year worldwide [13]. It is also known that 38–70% of female homicides are perpetrated by their current or former intimate partners, whereas 4–8% of male homicide are by their intimate partner [4, 14]. Despite its high prevalence or widespread prevalence, they are based on incomplete data collection systems in roughly half of the countries in the world [4, 12]. There is an evident need to identify a systematic method to gather data on the incidence of femicide that will allow comparisons across countries. Without reliable information, policy makers and programmers at all levels (national, regional or local) are unable to allocate resources so as to achieve the greatest impact in preventing these killings and reducing the harm they do to the victims and their relatives. Policy makers and programmers need information specific to their areas of concern [15].

In Europe, in 2011, the female death rate due to assault had great geographic variability, ranging from 0.03 deaths/100.000 inhabitants in Latvia to 2.85 deaths/100.000 inhabitants in Lithuania [16]. The heterogeneity in the surveillance systems makes it difficult to estimate the implications of these differences. Despite the fact that many countries have sex-disaggregated data on homicides, and a few (e.g. Spain and France)collect intimate partner-related homicide data in particular, the availability of specific data on all forms of gender-based homicide against women is far less developed [17]. Some of the challenges to accurate femicide surveillance across Europe include the misunderstanding of the gender basis of crimes and the limited available information in the existing homicide data registers about the relationship between victims and perpetrators, factors surrounding crimes, the motives of the perpetrators and the modus operandi [18]. At the time this study was conducted, only few national monitoring systems offered an example of how to overcome some of these challenges. The Finnish monitoring system, for example, is a registry of preliminary police investigations on intentional murders, manslaughters, killings, infanticides and negligent homicides that includes compulsory information related to nearly 90 variables regarding victim’s and offender’s characteristics, surrounding circumstances of the crimes, perpetrators’ behavior after the killing and spatial and temporal distribution [19].

Femicide is both a sociopolitical as well as a public health problem which has damaging effects on the lives of all women and their social environment, and holds negative implications for the whole society [20]. In order for Governments to begin to take action, there must be a common ground for understanding what femicide is, and the social costs, not only the individual ones, associated with it. If Governments were to understand this, not only will it help save lives, but it will also reduce the annual costs related to justice, social, welfare and social system [21]. Implementation of national surveillance systems on femicide and harmonization of these within Europe can facilitate a deeper understanding of this social and public health problem, and provide evidence for policy development, monitoring and prevention.

In 2013, the first professional and research network with focus on the issue of femicide was created within the platform of COST (European Cooperation in Science and Technology)[22]. The COST Action entitled “Femicide Action IS1206” brings together top-level experts on femicide and other forms of violence against women from 27 countries [23]. This is the first pan-European coalition on femicide involving researchers already studying the phenomenon nationally, with the purpose of advancing research clarity, agreeing on definitions, improving the efficacy of policies for femicide prevention, and publishing guidelines for the use of national policy-makers. The COST Action is organized in four working groups (WGs), and one of them (WG2) focuses on ‘Reporting’. The WG2 members organize annual meetings where data collection systems across Europe are analyzed, and data on femicide as well as on other related topics are compared and discussed. Discussions in the initial meeting of WG2 in early 2014 confirmed that most European countries collect data on homicides disaggregated by sex, which provides an overall picture of the magnitude of homicide against women across Europe. Furthermore, several countries collect data on the relationship between perpetrators and victims of homicide, and some of them collect data specifically on intimate partner homicide. However, it became clear that the collection of differentiated data on femicide was underdeveloped in most European countries.

The aim of this study on the COST Action on Femicide was to assemble expert opinions regarding strategies that might feasibly be employed to promote, develop and implement an integrated and differentiated femicide data collection system in Europe at both the national and international levels. The intended outcome of this process was to identify actions which are needed to improve the availability, collection and better comparability of data on femicide, taking into account different perceived needs across countries.

## Material and Methods

Concept mapping, an integrated mixed methods approach, was used to examine the diverse views of European experts on strategic actions needed to improve and systematize femicide data collection systems in Europe. The integration of qualitative and quantitative data occurs through sequential steps, beginning with generation of ideas (brainstorming), structuring of ideas through sorting and rating, representation of structuring in maps based in multivariate statistical methods, and finally collective interpretation of the maps [24]. This methodology was selected to meet the study objective based on its demonstrated usefulness in integrating the input of broad expert panels to guide development and planning, and its capacity to enable groups of actors to visualize their ideas around an issue of mutual interest and develop common frameworks [25, 26]. The participatory, structured nature of the concept mapping process was well-suited to the complexity of the task of integrating the views of femicide, Violence against Women (VAW), and data registration system researchers from different disciplines and European countries to develop policy recommendations for strategic action in the region. The fact that concept mapping combines qualitative input with multivariate analysis to produce a visual display of how a group views a particular topic was also considered in the selection of this method. Unlike purely qualitative techniques, concept mapping provides a structured approach for allowing participants to co-produce the content in focus in the study and interpret visual representations of their group perceptions [27].

The study was done with the approval of the members of the coordination board of the COST Action and written informed consent was asked of the participants. In addition, ethical approval for this study was granted by the Ethical Committee of the University of Alicante (Spain).

Concept mapping activities were carried out from December 2014 until February 2015 in three phases: 1) brainstorming, 2) sorting and rating, and 3) representation in maps and interpretation.

### Brainstorming

Based on discussions with the coordinators of the working group on ‘Reporting’, the researchers developed the following focus question to orient the brainstorming activity: *In what aspects shall we improve our own country’s data collection systems to collect accurate data on femicide*?

This question was sent via email, together with clear instructions of the entire concept mapping process to all of the 70 members of the whole COST Action. Participants were asked to write down as many strategies as possible in response to the question, with each strategy containing only one idea. The brainstorming phase was carried out from the 1^st^ of December, 2014 until the 13th of January, 2015. Twenty five members from 16 countries provided strategies, which the researchers checked to eliminate duplicates, and to divide complex strategies into simpler ones(Table 1). We were not able to recruit participants from Bosnia and Herzegovina, Denmark, Estonia, Finland, France, Iceland, Latvia, or Sweden.

**Table 1:** Participants in the brainstorming, sorting and rating by country of their institutions.

	Brainstorming	Sorting and rating
Spain	4	4
Israel	2	2
Italy	1	3
Belgium	0	1
Germany	1	2
Croatia	0	1
Lithuania	2	1
Macedonia	2	1
Malta	1	2
Poland	1	1
Portugal	1	1
Romania	1	6
Slovenia	2	1
UK	1	2
Austria	2	0
Greece	1	0
Netherlands	1	0
Cyprus	2	0
Total	25	28

Countries represented among participants in the different phases of the concept mapping study.

The researchers sent the refined list of 69 strategies to all participants to give them the opportunity to review and determine if their ideas were accurately reflected. In the next step, the strategies were entered in the Concept System software [28], which was used to facilitate the following steps of the process.

### Sorting and rating

The sorting and rating phase was accomplished in January 2015at the annual working group meeting in Rome. During the meeting, the first two authors presented the final list of strategies to the whole working group and explained the sorting and rating activities. Sorting activities consisted of experts organizing the strategies into meaningful groups, or thematic clusters, and giving them a title. Rating activities consisted of giving each strategy two ratings, for 1) its *relevance* to the goal of strengthening data collection systems and 2) its *feasibility* of being implemented [29]. Each strategy should be given a value from 1 to 6 for relevance and feasibility, where 1 meant very low relevance or feasibility and 6 meant very high relevance or feasibility. Experts who did not finish the sorting and rating in Rome or who were not present were able to complete the sorting and rating online. Of the 45 experts invited to participate in the sorting and rating, 28 from 14 countries, provided answers (Table 1).

### Representation in maps and interpretation

In the representation and interpretation step, the gathered data was analyzed using concept mapping techniques that facilitate visualization of thematic clusters, and identification of areas of consensus for action. The sorting data was analyzed using multi-dimensional scaling to generate point maps, where strategies are plotted in a two-dimensional graph based on a similarity matrix, which captures the number of times experts grouped them together. Strategies that were more frequently sorted together are positioned closer to each other. The degree of fit between the point map and the data in the similarity matrix is reflected by the stress index, where a lower value indicates a better fit. The stress index of the final point map, which was used as the base for identifying clusters, was 0.207. This score was in line with the results of other concept mapping studies, as reflected by the results of a meta-analysis of concept mapping projects, which estimated an average stress value of 0.285 with a standard deviation of 0.04 [24].

Hierarchical cluster analysis was used to show options for aggregating strategies into clusters based on their proximity to each other in the point maps. Cluster maps ranging from seven to twelve clusters were evaluated through discussion among the researchers by reviewing the strategies grouped together at successive levels of clustering based on their conceptual coherence and the value of the precision offered at each level. For example, in moving from the level of nine to ten clusters, the clusters “Public Awareness” and “Media coverage” were separated, and the researchers decided this division was valuable in distinguishing the strategies. Ten clusters were identified as the final solution, and names were assigned by the researchers through consideration of the content of the clusters and the group names suggested by experts. This level of division was similar to the average number of thematic clusters identified in the sorting activity, where participants created an average of nine clusters (average = 8.8 clusters, standard deviation = 4.2, minimum = 4, maximum = 27).

Rating data was analyzed to identify the priorities for action based on the views of the group as a whole, as well as dynamics in the perception of priorities across regional sub-groups using role-stratified averages. We have a variety of countries in the sample, and we wanted to explore whether the level of general development of the country could influence the perceived relevance of the strategies proposed. The rationale behind this assumption was that in countries with better public systems, registers in general and also those related to femicide would be better and so the priorities might differ from those of countries with less developed registers and public institutions. We choose the Human Development Index(HDI) as a proxy for this. The HDI measures the average achievements in a country in three dimensions of human development: health dimension, measured by life expectancy at birth; education component, measured by adult literacy rate and the combined primary, secondary and tertiary gross enrollment ratio; the standard of living, measured by Gross Domestic Product per capita in purchasing parity [30]. The scores for the three HDI dimensions are then aggregated into a composite index using geometric mean. This index score was used to classify countries into two groups due to its association with different mortality causes [31–33], diseases distribution [34, 35] and health behavior [36, 37]. Seven countries with higher HDI (Germany, Israel, Belgium, Slovenia, Spain, Italy and the UK) were included in one group and seven countries with lower HDI (Malta, Poland, Lithuania, Portugal, Croatia, Romania and Macedonia) were included in the other group.

After creating the maps, initial results of these analyses were discussed during the second part of the working group meeting in Rome to facilitate their interpretation. During the meeting, the participants worked in small groups to evaluate the appropriateness of the clusters generated by the analysis and were asked to determine if the strategies within each cluster were coherent, if clusters could be joined or divided and if the titles were appropriate. Experts also reflected on their own experiences to improve data collection systems and discussed connections among the clusters of actions. Notes from these discussions, as well as on-going dialogue among the core research team, provided the base for finalizing the cluster map and identifying domains of thematically related clusters.

## Results

### Actions to promote improved data collection systems

**Fig 1:**
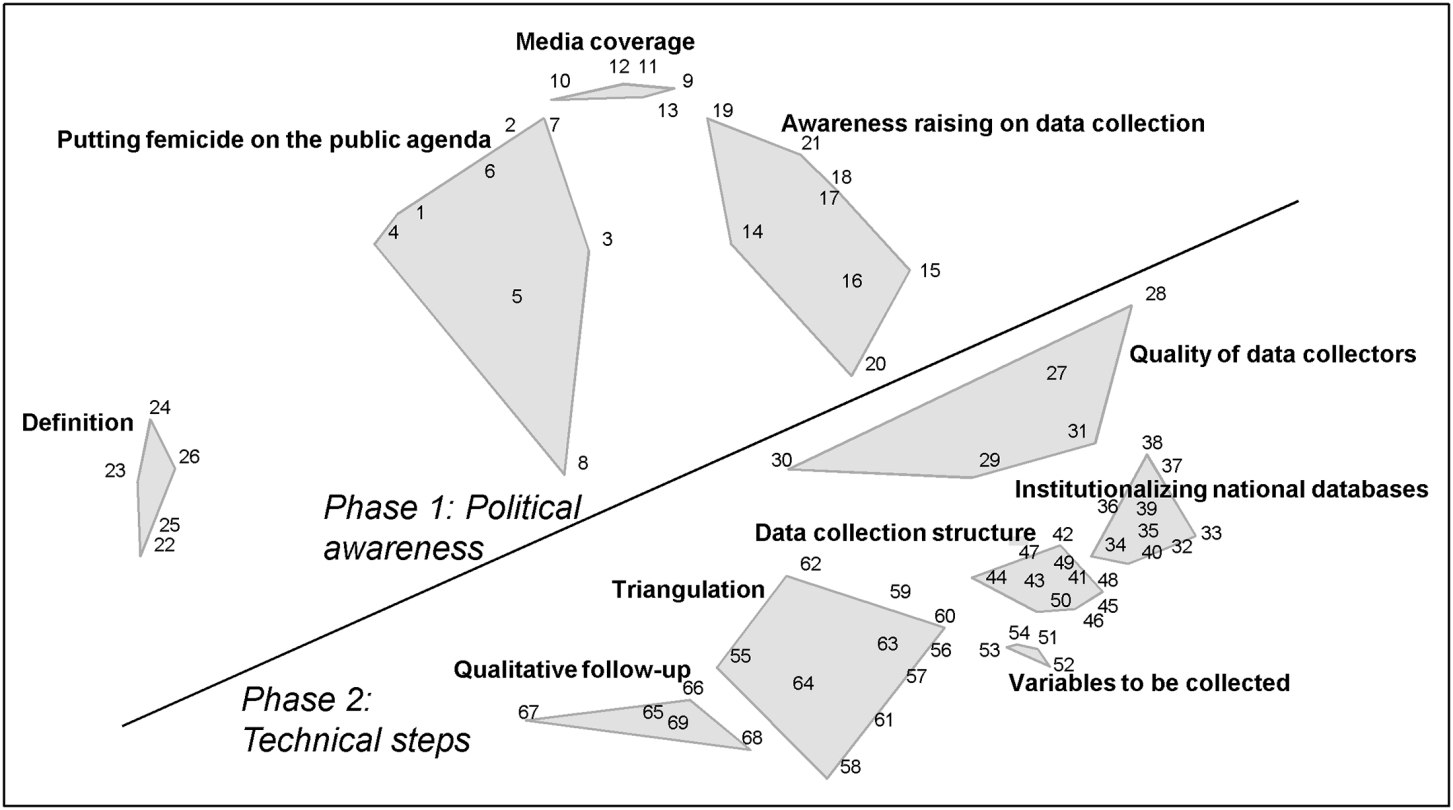
Clusters of actions to promote improvement of data collection systems on femicide across Europe. Cluster map based on experts’ thematic grouping of action strategies.

Of the 69 strategies that were generated by the participants, the final clustering solution identified 10thematic areas of action: “Putting femicide on the public agenda”, “Media coverage”, “Awareness raising on the importance of data collection”, “Definition”, “Quality of data collectors”, “Institutionalizing national databases”, “Data collection structure”, “Variables to be collected”, “Triangulation”, and “Qualitative follow-up” (Fig 1).

Based on relationships among these clusters, the cluster map was divided into two domains: “Political awareness” and “Technical steps”. The six clusters in the domain “Technical steps” include straightforward actions that would directly improve the content, structure, quality and continuity of national data collection systems, as well as measures to improve detection and follow-up investigation. The four clusters in the domain “Political awareness” include actions directed towards enhancing public awareness of the existence and scale of femicide, as well sensitizing key groups to their role in data collection on femicide, and clarifying a common understanding of what constitutes femicide. Table 2 presents an overview of the content of the clusters and selected strategies (for a complete list of all strategies see S1 File).

**Table 2:** Overview of the content of the clusters, selected strategies and average ratings of clusters items.

CLUSTERDESCRIPTION	SELECTED STRATEGIES	RELEVANCE	FEASIBILITY
PHASE 1. POLITICAL AWARENESS			
Putting femicide on the public agenda—Strategies to enhance political will in order to make femicide a public priority.	- Ensuring political will and commitment (1). Putting the concept of femicide into the academic, social, political and legal agenda (4).	5.05	4.29
Media coverage- Strategies to improve the quantity and quality of media coverage of the problem of femicide.	- Publicizing the information on femicide through accessible communication venues (9). Training journalists on how to report these cases properly (10).	4.70	4.44
Awareness raising on data collection—Strategies to raise awareness of the importance of collecting accurate data on femicide among data collectors and stakeholders.	- Alerting the public institutions, ministries and other state authorities to the need to identify, register and analyze the characteristics of femicide as a specific crime (14). Increasing awareness among data collection personnel (15).	4.88	4.40
Definition—Strategies to reach consensus on a definition of femicide that captures the complexity of the phenomenon.	Establishing a clear definition of femicide across countries (22).	4.69	4.04
PHASE 2. TECHNICAL STEPS			
Quality of data collectors—Strategies to improve the quality of the data collection systems on femicide, with special focus on adequate sensitization and training of professionals involved in the collection and reporting of data.	Training those in charge of collecting those data on the importance of gathering correct information on all relevant aspects (28).	5.10	3.74
Institutionalization of national data base—Strategies to ensure that countries have a publicly funded, centralized and sustainable data collection system on femicide.	- Establishing a database, publicly funded and sustained, to collect information on all forms of violence against women including femicide (32). Developing a centralized system that gathers data from all relevant institutions (34).	5.28	4.12
Data collection structure—Strategies to ensure the quality of data collection systems of femicide, with focus on structural and organizational aspects.	- Standardizing data collection systems across police and court data collection system (42). Deciding on what information to collect based on the state of the art of the issue (50).	4.66	3.90
Variables to be collected—Suggestions for specific, standardized information to be gathered for every case of a female homicide.	- Collecting basic socioeconomic data on victims and offenders, including their age, education level, employment status and/or occupational class, place of birth, and area of residence (11). Ensuring that all types of data collection systems (crime, court, etc.) collect at least the following information: sex of both victim and perpetrator, type of relationship between them, prior history of domestic violence, previous institutional interventions (51).	5.20	4.21
Triangulation—Strategies to enhance triangulation across data collection systems, both at the national level, to enhance case detection, and at the regional level, to enhance comparability.	- Identifying a minimum set of variables covered that allow us to know the situation in Europe and make comparisons between countries (55). Triangulating monitoring systems data with newspaper articles, police and court statistics (59).	4.69	4.08
Qualitative follow up—Strategies to collect in depth information on every suspicious case in order to diminish underreporting and better understand the phenomenon.	- Developing qualitative research on motives, context and background of the cases in order to find out, if and how these crimes could be prevented (65).	4.56	4.11

Description of the clusters depicted in Fig 1. Examples of strategies and the average rating of strategies within the cluster. Corresponding numbers of example strategies are indicated in parenthesis.

The varying size of the clusters depicted in the map reflects the tightness of the conceptual coherence of the strategies the clusters contain, while the proximity of clusters reflects perceived relationship between the strategies they contain. The relatively larger size of the clusters “Putting femicide on the public agenda” and “Raising awareness on the importance of data collection” reflects the diversity in the nature of actions needed to generate public consciousness and shift political will. While the concentration of many strategies in the smaller and closely proximal clusters, “Variables to be collected”, “Quality of data collection structure”, and “Institutionalizing national databases”, reflects that the technical steps required to strengthen data collection systems are numerous and closely interrelated.

### Identifying priority actions

Analysis of the overall average ratings of the items that make up the clusters indicated that the cluster “Institutionalizing national databases” was the most relevant (5.28), followed by “Variables to be collected” (5.20) and “Quality of data collectors” (5.10). The clusters “Qualitative follow up” (4.56) and “Data collection structure” (4.66) were rated with the lowest relevance. It is also noteworthy that items in the cluster “Definition” received a relatively low overall rating (4.69). The ratings on feasibility were overall lower than those of relevance. The clusters with the highest rating on feasibility included “Media coverage” (4.44), “Awareness raising on data collection” (4.40) and “Putting Femicide on the Public Agenda” (4.29), while the lowest rated were “Quality of data collectors” (3.74), “Data collection structure”(3.90), and “Definition” (4.04) (Table 2 and S1 File).

Strategies with the highest ratings for both relevance and feasibility (items 51, 55, 32, 33, 28) referred to ensuring that specific types of information are collected in a standardized and institutionalized way that provides a base for identifying cases and monitoring femicide cases at a regional level. The strategies with the lowest ratings for relevance and feasibility referred to strategies to conduct in-depth analysis of suspected cases of femicide (items 49, 64, 69, 63, 48) (Table 3).

**Table 3:** Summary of the most and the least relevant and feasible strategies to build femicide data collection systems according to participants’ opinions.

The 5 most relevant and feasible strategies	The 5 least relevant and feasible strategies
51. Ensuring that all type of data collection systems (crime, court, etc.) gather at least the following information: sex of victim and perpetrator, type of relationship between them, prior history of domestic violence and previous institutional interventions.	49. Ensuring that cases where the court does not have enough evidence to convict the offender for a crime likely to be femicide are included in the monitoring systems as suspicious cases of femicide.
55. Identifying a minimum set of variables covered at least in the European context that allow us to know the situation in Europe and make comparisons between countries.	64. Reviewing past cases of women murdered to identify if they are femicides or not.
32. Establishing a database, publicly funded and sustained, to collect information on all forms of violence against women including femicide.	69. Interviewing perpetrators, relatives, friends, neighbors and acquaintances.
33. Ensuring that national data on femicide are collected following international recommendations and comparable with data collected in other countries.	63. Tracking cases in which the perpetrator commits suicide after committing the intimate partner femicide.
28. Training those in charge of collecting those data on the importance of gathering correct information on all relevant aspects.	48. Upgrading national records about the deaths and causes of death with the information about murder as a cause of death (Ministry of Health) and using this source as a possible detector of those murders that are committed before the perpetrator commits a suicide.

Results of strategies rating based on experts’ assessment of relevance and feasibility.

### Analyzing differences in priorities across countries

**Fig 2:**
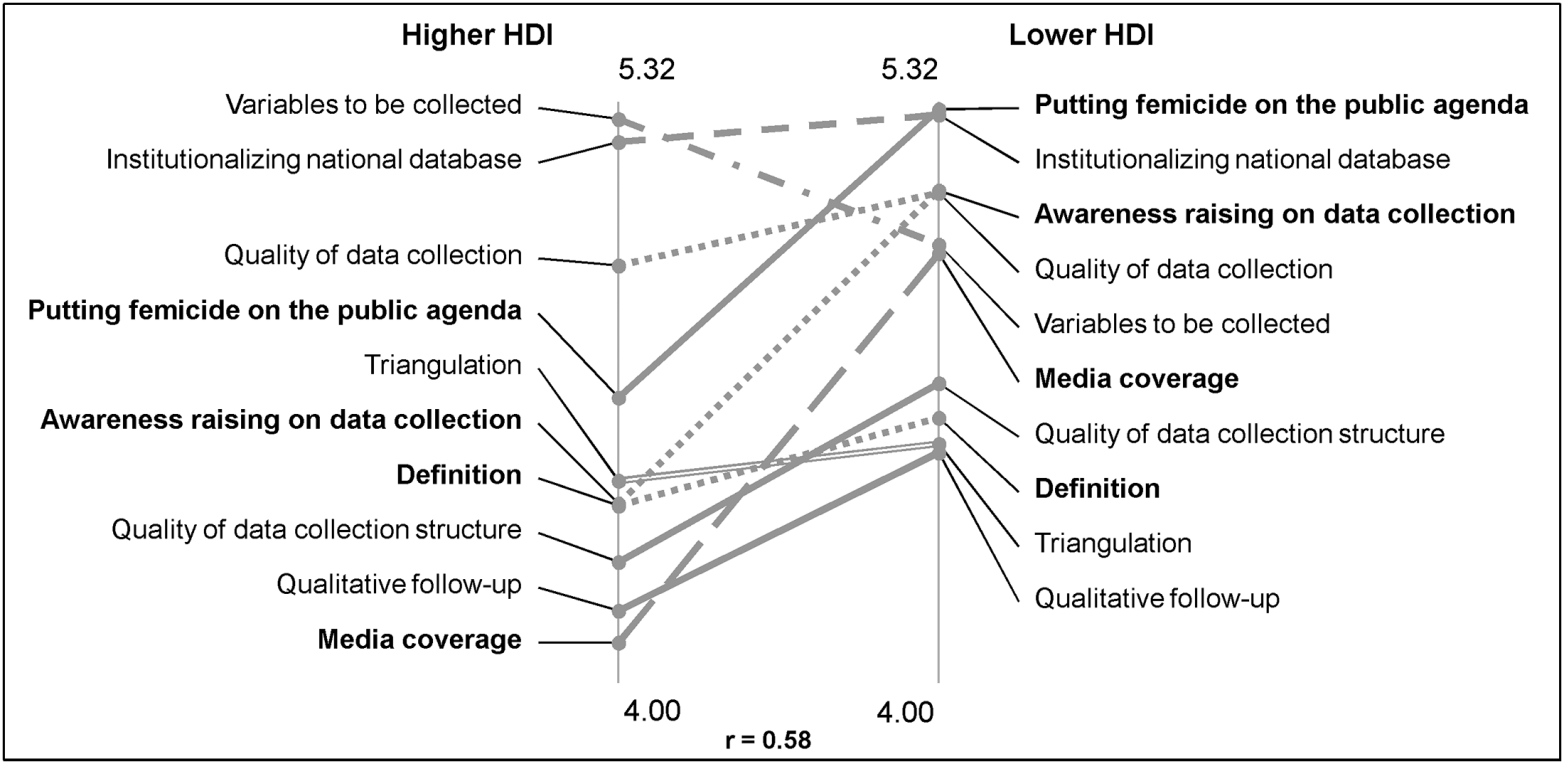
Comparison of average cluster relevance ratings by countries with higher and lower HDI. The clusters with significant difference in the domain of “Political awareness” are highlighted in bold.

The comparison of average rating of the relevance of action clusters showed differences between the priorities of countries with lower and higher HDI ranking. Countries with lower HDI rated clusters in the domain of “Political action” most highly, whilst the countries with higher HDI gave highest rating to clusters in the domain of “Technical steps”. The greatest difference in the perceived relevance across groups was found in the clusters “Putting femicide on the public agenda”, “Awareness raising on data collection”, and “Media coverage”, and t-tests showed that these difference were significant with t-values, degrees of freedom and levels of significance of(-3.01, 14, p<0.01),(-3.59, 14, p<0.05), (-5.18, 8, p<0.001), respectively. Both groups agreed on the lower relevance of clusters on “Qualitative follow up”, “Triangulation”, “Data collection structure” and “Definition”. Both groups also agreed on the very high relevance of “Institutionalizing national databases”. Actions belonging to the “Media coverage” cluster were rated with the lowest relevance by experts from countries with higher HDI, and among the most relevant by those from countries with lower HDI. Despite these variations in perceived relevance of actions, the sub-groups’ assessments of feasibility were very similar (Fig 2).

## Discussion

The strategies generated through the brainstorming step provided a broad range of recommendations that may be applied within and across different countries in order to improve the availability, collection and monitoring of femicide data. These include political will, technical specific requirements and the involvement of different actors—governments, mass media, police bodies, courts and professionals, who are in charge of identifying, registering and monitoring. Priority clusters of actions were also identified within this range of strategies, and according to experts’ assessment, “Institutionalizing national databases” was found to be most relevant, while “Media coverage” was rated most feasible. Variation in ratings across countries indicated differing perceptions of priorities based on their current situation.

As has been observed in relation to other health information system issues [38, 39], the experts’ responses reflected that promoting and implementing concrete changes requires not only technical steps, but also socio-political processes. Strategies related to the latter emerged despite the explicit emphases on data collection on femicide in the initial focus question. The connection between technical change and socio-political processes is also reflected in the interrelationship among actions related to institutionalizing national databases, defining the variables to be collected and the quality of data collectors. In order for registers to function continuously, they have to be institutionalized [40], which depends on political will backed by adequate state funding. When registers are institutionalized, the definition of indicators is enabled and priority is given to certain indicators [41] that will enable differentiation of several forms of femicides from women´s homicides [42]. The definition of variables and indicators, as it has been shown with the implementation of health information systems [39] or in the harmonization of European registers [43], is strongly connected to the ability to collect valid and reliable data describing both the extent of its occurrence, its context and background of risk factors to establish action lines for its prevention.

Feasibility ratings were lower overall than those of relevance. Experts in this study may have clear ideas about actions needed to ensure improved data collection on femicide (relevance), but they also have experience in the difficulties of achieving these changes (feasibility). Challenges include existing structures of policies and data collection systems, where those responsible may not see the importance of collecting this kind of data, or lack the necessary training, budget or statistical data collection systems to do so. The difficulty of getting different agencies to cooperate with each other to produce this data is another important challenge that is reflected in the low feasibility scores given to “Definition”. Despite the relatively high perceived relevance of actions to reach consensus on a definition of femicide, experts are aware that it will be very difficult to harmonize definitions and also to have enough information for each case of female homicide to allow the data collectors to define it as femicide.

In countries with lower HDI ranking, “Political action” was considered the most relevant and necessary first step. In countries with higher HDI ranking, the “Technical steps” were more relevant. This result could perhaps be explained by the observation that in countries with lower HDI, topics of violence against women or femicide are not prioritized in policies or public discourse. In Portugal, for example, as well as in most East-European countries, women civil society groups addressing VAW are more recently formed than those located in, for example, UK where women’s groups and public awareness about this problem started in the early 1970s [14]. Based on this pattern, it is expected that experts from the former group of countries would perceive the relevance of actions to heighten public awareness and strengthen the political will for data collection activities more strongly than experts from the latter ones.

The results of this study should be interpreted taking into account several study limitations. The final samples of experts in both, brainstorming and sorting/rating steps do not fully represent all countries of the European region. Despite the fact that we asked 70 professionals from all countries involved in the COST Action “Femicide across Europe”, only a part of them accepted this invitation. We probably ended up engaging those participants that were more interested in the topic. Unfortunately, it was not possible to recruit a representative from Finland or France, where examples of femicide-related intimate partner violence and domestic violence data registries have been further developed [17, 19]. It would be important to gain their perspectives in future research. We conducted this concept mapping study with the members of the COST Action due to their professional experience in the topic of femicide, but this expertise not always was focused in data collection systems. However, this profile may be also considered as strength due to their understanding of issues surrounding the development of femicide data collection systems (such as those related to political action) as well as those related to technical aspects. The ecological perspective of this study limits the transferability of our results to the specific situation of each country. Future research should be applied within the specific contexts of each country.

Among the strengths, the use of the concept mapping method must be highlighted, as it allowed us to structure and rate relevant aspects of a complex topic in a very short time and to integrate various expert opinions from different countries. It thus contributed directly to clear and manageable scientific results that may provide an important basis for improved data collection systems. The timing of the study is also strength. Until the establishment of the COST Action IS1206 “Femicide across Europe” in mid- 2013, European agencies had never recognized the lethal act of femicide as a separate topic, although they had funded initiatives on gender and violence where femicide was a rather side-topic. Nowadays with COST Action IS1206 operating for the past two years, the phenomenon of femicide in Europe is entering the public agenda, and intermeshing with that of global institutions such as ACUNS(Academic Council on the United Nations System) and EIGE(European Institute for Gender Equality)[3].

In conclusion, the results of this study provide a concrete plan of the next (political and technical) steps to be taken in order to improve data collection and monitoring on femicide in and across European countries: Institutionalizing a national database on femicide, agreeing on a minimum set of variables that have to be collected in each case, and investing in the training of those professionals who are in charge of collecting the data. Furthermore, expert assessments revealed that implementing and sustaining femicide data collection systems entails not only technical data collection, but also a firm political commitment. Once in place, the evidence produced can contribute to increased public awareness and demand for a public health sector response, as done with IPV, as well as providing concrete information on risk factors and risk groups to guide police, legal, educational, and political forces in development of prevention strategies and services.

## Supporting Information

S1 File Complete list of strategies with individual ratings, organized by cluster. (DOCX)
