# Correction: Expert Opinions on Improving Femicide Data Collection across Europe: A Concept Mapping Study

**DOI:** 10.1371/journal.pone.0154060

**Published:** 2016-04-14

**Authors:** Carmen Vives-Cases, Isabel Goicolea, Alison Hernández, Belen Sanz-Barbero, Aisha K. Gill, Anna Costanza Baldry, Monika Schröttle, Heidi Stöckl

The eighth author’s name is spelled incorrectly. The correct name is Heidi Stöckl.

There is an error in the fourth sentence of the penultimate paragraph in the Discussion section. The correct sentence is: Femicide was, for a long time, only addressed in Europe under the wider umbrella of violence against women. It gained greater research and public attention by European and global projects and institutions only in the last decade, under the COST Action IS1206 on Femicide, the EU Daphne Justice Programmes and addressed through ACUNS (Academic Council on the United Nations System) [44].

This updated sentence cites a new reference, which is: Weil, S. “Femicide in Europe”. In: Dimitrijevic, M., Filip, A and Platzer M (eds) Femicide: a Global Issue that Demands Action. Taking Action against Gender-Related Killing of Women and Girls, Vol. 4. Vienna: ACUNS; 2015. Pp.118-121.
