# Supplementary material for: Expert Opinions on Improving Femicide Data Collection across Europe: A Concept Mapping Study
Source: PLoS One. 2016 Feb 9;11(2):e0148364. doi: 10.1371/journal.pone.0148364 (PMC4747603; doi:10.1371/journal.pone.0148364)
Supplement: S1 File — (DOCX) [file pone.0148364.s001.docx]

| **S 1 File. Complete list of strategies with individual ratings, organized by cluster** | | | | | | |
| --- | --- | --- | --- | --- | --- | --- |
|  | | |  | **Relevance** | **Feasibility** | |
| **Putting femicide on the public agenda** | | | | **5,05** | **4,29** | |
|  | 1 | Ensuring political will and commitment. | | 5,36 | 3,43 |  |
|  | 2 | Raising awareness among police officers investigating a potential femicide on what it is and how it can be detected, e.g. what clues to look for and what to ask. | | 5,25 | 4,46 |  |
|  | 3 | Produce a special report on femicides, as is done in Spain and France. | | 5,23 | 4,56 |  |
|  | 4 | Putting the concept of femicide into the academic, social, political and legal agenda | | 5,21 | 4,54 |  |
|  | 5 | Fostering the collaboration between researchers and institutions which work on the field (police, social services, NGO's, courts,...). | | 5,07 | 4,39 |  |
|  | 6 | Strengthening the discussion about murder against women within the violence-against-women context. | | 4,93 | 4,71 |  |
|  | 7 | Training practitioners (inside and outside of the academic filed) on the meaning, definition of femicide and importance of its use. | | 4,75 | 4,71 |  |
|  | 8 | Using legislation process as a primary tool to compel the State to gather the statistics on femicide. | | 4,57 | 3,50 |  |
| **Media** | |  | | **4,70** | 4,44 |  |
|  | 9 | Publicizing the information on femicide through accessible communication venues. | | 4,82 | 4,86 |  |
|  | 10 | Training journalists on how to report properly these cases. | | 4,75 | 4,14 |  |
|  | 11 | Ensure that media is getting hold of reports on femicide to encourage wider publication. | | 4,69 | 4,60 |  |
|  | 12 | Raising awareness among journalists on these issues. | | 4,68 | 4,29 |  |
|  | 13 | Promoting the use of the word femicide in public and media reporting. | | 4,54 | 4,32 |  |
| **Awareness raising on data collection** | | | | **4,88** | **4,40** |  |
|  | 14 | Alerting the public institutions, ministries and other state authorities to the need to identify, register and analyze the characteristics of femicide as a specific crime. | | 5,11 | 4,11 |  |
|  | 15 | Increasing awareness among data collection personnel (police, court etc) about femicide and what it means. | | 5,11 | 4,32 |  |
|  | 16 | Including the reporting of femicide in the Official report of the Ministry of internal affairs on homicide. | | 5,07 | 4,25 |  |
|  | 17 | Disseminating collected information on femicide among the most relevant institutions. | | 5,00 | 4,79 |  |
|  | 18 | Presenting regularly information on femicide and all other forms of violence against women in official police statistics. | | 4,96 | 4,32 |  |
|  | 19 | Disseminating femicide information gathered through the data collection system so that data collectors are aware of the relevance of their work. | | 4,75 | 4,39 |  |
|  | 20 | Presenting case studies of femicides in the context of data collection (Police stations, Central Statistical Service of the Police in each country) that summarize the problem to convince of its relevance. | | 4,68 | 4,21 |  |
|  | 21 | Making a conference on data collection improvements. | | 4,32 | 4,79 |  |
| **Definition** | | | | **4,69** | 4,04 |  |
|  | 22 | Establishing a clear definition of femicide across countries | | 5,39 | 4,29 |  |
|  | 23 | Ensuring that femicide is defined as consequence of gender based violence - as an extreme manifestation of male domination against women. | | 4,93 | 4,32 |  |
|  | 24 | Introducing the term femicide clearly in the Penal Code. | | 4,54 | 3,07 |  |
|  | 25 | Typifying different kinds of femicide properly according its definition as a "Result of Domestic Violence and Intimate partner violence; Killing of Women and Girls in the Name of Honour; Dowry-related Femicide; Organized Crime related Femicide ; Targeted Killing of Women at War; Female Infanticide and Gender-Based Sex-Selective Foeticide ; Genital Mutilation Related Femicide and the Killing of Women due to the Accusation of Witschcraft/Sorcery ; The Misogynyst Slaying of Women" | | 4,32 | 4,43 |  |
|  | 26 | Avoiding narrowing the understanding of femicide as a crime only committed by males - but also as crimes committed by women in the name of patriarchal ideology (ex: women in some cultures who kill their female babies or commit foeticide, as a consequence of patriarchal ideology). | | 4,25 | 4,07 |  |
| **Quality of data collectors** | | | | **5,10** | **3,74** |  |
|  | 27 | Improving the financial support for data collection on femicide. Securing specific budget to be set aside to achieve the tasks outlined | | 5,39 | 2,93 |  |
|  | 28 | Training those in charge of collecting those data on the importance of gathering correct information on all relevant aspects. | | 5,36 | 4,57 |  |
|  | 29 | Ensuring a system that requires police officers or homicide officers to fully complete all the information before a case can be closed to reduce missing information | | 5,25 | 3,82 |  |
|  | 30 | Simplifying access to police data and data from social services for researchers - not only statistical data but also access to personal records with contextual information. | | 4,86 | 3,39 |  |
|  | 31 | Giving feed-back on the quality of the data collected to individuals and institutions collecting data. | | 4,64 | 4,00 |  |
| **Institutionalization of national database** | | | | **5,28** | **4,12** |  |
|  | 32 | Establishing a data-base, publicly funded and sustained, to collect information on all forms of violence against women including femicide. | | 5,68 | 4,29 |  |
|  | 33 | Ensuring that national data on femicide are collected following international recommendations and comparable with data collected in other countries. | | 5,50 | 4,29 |  |
|  | 34 | Developing a centralized system that gathers data from all relevant institutions | | 5,36 | 4,04 |  |
|  | 35 | Establishing an electronic system to collect data. | | 5,25 | 4,46 |  |
|  | 36 | Ensuring timely maintenance of the homicide monitoring system. | | 5,21 | 4,04 |  |
|  | 37 | Monitoring the quality of the data collection system. | | 5,21 | 4,18 |  |
|  | 38 | Implementing mandatory monitoring systematic data collection in accordance with the scope of acceptable definition of femicide. | | 5,18 | 3,64 |  |
|  | 39 | Ensuring official-governmental data collection systems to gather and map femicide-related information. | | 5,11 | 4,07 |  |
|  | 40 | Establishing a unified system to collect data from each of the regions in each country. | | 5,04 | 4,07 |  |
| **Data collection structure** | | | | **4,66** | **3,90** |  |
|  | 41 | Building a specific register of femicide, with specific variables and categories, that may include some of the variables collected in the mortality registers. | | 5,46 | 4,07 |  |
|  | 42 | Standardizing data collection systems across police and court data collection system | | 5,36 | 3,82 |  |
|  | 43 | Ensuring that records are individualized, therefore the information collection unit must be the murdered woman. | | 5,04 | 4,04 |  |
|  | 44 | Collecting data about cases of murder-suicide and/or women's suicide as a form of responding to exposure to violence and, consequently, as a form of femicide. | | 4,57 | 4,11 |  |
|  | 45 | Registering national cases of gender-based killings (femicide) by geographical area. | | 4,54 | 4,50 |  |
|  | 46 | Establishing a special data collection system on femicide and immigrant women. | | 4,46 | 3,64 |  |
|  | 47 | Aggregating data from NGO's with the data of the police authorities. | | 4,43 | 4,43 |  |
|  | 48 | Upgrading national records about the deaths and causes of death with the information about murder as a cause of death (Ministry of Health) and using this source as a possible detector of those murders that are committed before the perpetrator commits a suicide. | | 4,29 | 3,61 |  |
|  | 49 | Ensuring that cases where the court does not have enough evidence to convict the offender for a crime likely to be femicide are included in the monitroring systems as suspicious cases of femicide. | | 4,25 | 2,71 |  |
|  | 50 | Deciding on what information to collect based on the state of the art of the issue. | | 4,21 | 4,11 |  |
| **Variables to be collected** | | | | **5,20** | **4,21** |  |
|  | 51 | Ensuring that all type of data collection systems (crime, court, etc) collect at least the following information: sex of both victim and perpetrator, type pf relationship between them, prior history of domestic violence and previous institutional interventions. | | 5,61 | 4,68 |  |
|  | 52 | Collecting basic socioeconomic data on victims and offenders, including their age, education level, employment status and/or occupational class, place of birth, length of stay (if applicable), administrative status (if applicable) and other characteristics that may be relevant such as having or not a physical or mental disability or area of residence. | | 5,18 | 4,29 |  |
|  | 53 | Ensuring that all type data collection systems (crime, court, etc) collect as well information related to: length of the relationship; children in common; and if the victim was or not pregnant when she was murdered. | | 5,04 | 3,89 |  |
|  | 54 | Gathering information from each registered femicide case about: date and time of aggression; form of being murdered (gun, knife, hit, choked etc ..,); place where aggression occurs; if other people were present or not during the aggression; if the aggression was made by one person or more than one; and whether the aggression occurs being the victim and / or perpetrator under the influence of alcohol and / or other drugs or not. | | 4,96 | 4,00 |  |
| **Triangulation** | | | | **4,69** | 4,08 |  |
|  | 55 | Identifying a minimum set of variables covered at least in the European context that allow us to know the situation in Europe and make comparisons between countries. | | 5,57 | 4,57 |  |
|  | 56 | Establishing collaborations with women NGOs, especially shelters, about the data collection to receive additional information. | | 5,11 | 4,82 |  |
|  | 57 | Linking femicide statistics with intimate partner violence statistics â€“ they seem to be reported at different webpages and by different departments, but actually they should be combined and shown in the same reports | | 5,00 | 4,24 |  |
|  | 58 | Collaborating with neighboring countries or other statistical offices to ensure that indicators collected are comparable. | | 5,00 | 3,96 |  |
|  | 59 | Triangulating monitoring systems data with newspaper articles, police and court statistics. | | 4,82 | 4,25 |  |
|  | 60 | Registering former hospitalization and injury reports of women who show signs of prior abuse. | | 4,68 | 3,61 |  |
|  | 61 | Linking criminal reports to mental Health and Medical reports of perpetrators and potentially victims | | 4,46 | 3,72 |  |
|  | 62 | Monitoring newspapers articles to gather detailed information in addition to police and court statistics. | | 4,25 | 4,39 |  |
|  | 63 | Tracking cases in which the perpetrator commits suicide after committing the intimate partner femicide. | | 4,04 | 3,71 |  |
|  | 64 | Reviewing past cases of women murdered to identify if they are femicides or not | | 3,96 | 3,46 |  |

| **Qualitative follow up** | | | **4,56** | **4,11** |
| --- | --- | --- | --- | --- |
|  | 65 | Developing qualitative research on motives, context and background of the cases in order to find out, if and how these crimes could be prevented. | 4,75 | 4,64 |
|  | 66 | Performing qualitative in-depth studies of court cases to collect contextual information of femicides: what lead to it, what beliefs does the perpetrator have, how does the victim come in. | 4,71 | 4,39 |
|  | 67 | Promoting qualitative research of homicides and suspicious deaths of women to determine if certain homicide of women can be qualified as femicide. | 4,61 | 4,54 |
|  | 68 | Ensuring consistent follow up of each perpetrator of femicide - whether he ends up committing or attempting suicide; whether he ends up or not in prison; verdict and penalty. | 4,57 | 3,46 |
|  | 69 | Interviewing perpetrators, relatives, friends, neighbors and acquaintances. | 4,14 | 3,50 |
